# Pulmonary mucormycosis after renal transplantation: A case report and a literature review

**DOI:** 10.1016/j.amsu.2022.103889

**Published:** 2022-06-01

**Authors:** Ekhlass Abu Asabeh, Zahraa M.M. Zeer, Osama N. Dukmak, Mohammad Eid Al Mohtasib, Yousef Abu Asbeh

**Affiliations:** a Faculty of Medicine, Al-Quds University, Palestine; b Thoracic Surgery Department, Al-Ahli Hospital, Palestine

**Keywords:** Mucormycosis, Renal transplantation, Immunosuppression, Case report, Covid-19, Fungal infection

## Abstract

Mucormycosis is a rare fungal infection mostly encountered in immunosuppressed patients. Other major risk factors are diabetes mellitus, solid organ transplant and chronic use of glucocorticoids. Early diagnosis should be obtained as soon as possible due to the infection's aggressive behavior and high probability of dissemination. Here we present a case of pulmonary mucormycosis in a non-diabetic patient, known to have systemic lupus erythematous and had a renal transplant recently presented with shortness of breath and was treated with antibiotic as a case of chest infection with minimal improvement. Then, after full investigations, he seemed to have mucormycosis which was successfully treated with combined liposomal amphotericin B and resection of the infectious lesion.

## Highlights

• Mucormycosis is a highly lethal medical condition that occurs more often in immunocompromised patient.
• Proper medical therapy should be started immediately when this infection is suspected.
• The overall mortality rate of Pulmonary mucormycosis is 76%, increasing to 95% if extrathoracic dissemination occurs.
• Survival of more than two weeks in the absence of treatment is extremely rare.

## 1 Introduction

Mucormycosis is a rare fungal infection caused by fungus of the Mucorales order [1]. It's known to be an aggressive opportunistic angio-invasive fungal infection that, despite early detection and intensive treatment, has a significant morbidity and mortality rate. Mucormycosis has been connected to a variety of medical conditions, with diabetes mellitus being the most common underlying disease [2]. Immunosuppression, solid organ transplant, hematological malignancies, autoimmune illnesses, iron overload are all key risk factors, and this infection has recently been noticed in covid-19 patients as well [3].

Mucormycoisis incidence after renal transplant recipient is approximately 0.4–0.5 per 1000 patients [4] and pulmonary involvement seemed to be the second most common site for infection after renal transplant [5]. Most of these infections occur in the first two months after transplant with a high mortality rate reaching about 76% [6]. In pulmonary mucormycosis, clinical diagnosis is challenging, and early detection is critical for this life-threatening infection [7]. Treatment involves combined surgical debridement and parenteral liposomal amphotericin-B along with modification of the underlying disease [1].

## 2 Case presentation

A 46-year-old male patient, who had received a renal transplant three months earlier, presented with shortness of breath occurred even when he was at rest, increased by minimal exertion for a period of one week associated with persistent productive cough of whitish sputum, he had previously experienced similar episodes, which were misdiagnosed as a chest infection and treated with antibiotics with mild improvement.

His past medical history includes chronic kidney disease, where he had been on regular dialysis over the past eight years, he also has systemic lupus erythematosus, antiphospholipid syndrome, peripheral vascular disease, lower limbs deep vein thrombosis, transient ischemic attack, coronary artery disease, and ten days after his kidney transplants, he underwent cardiac catheterization twice with stent insertion. He also had a 30-pack- year smoking history.

The patient was on Nystatin, Tacrolimus, Prednisolone, Rivaroxaban, Clopidogrel, Aspirin, Atorvastatin, Bisoprolol, Furosemide, Valganciclovir, sulfamethoxazole-trimethoprim, Ceftriaxone.

Physical examination revealed an ill appearing man, afebrile and he was on 5 L oxygen therapy. Bilateral lower zone crepitations were present with diminished air entry over the right lower chest. The reminder of the physical examination was non contributory.

His laboratory values were significant for normocytic anemia and elevated creatinine with a baseline level of 1.7 mg/dl.

**Fig. 1:**
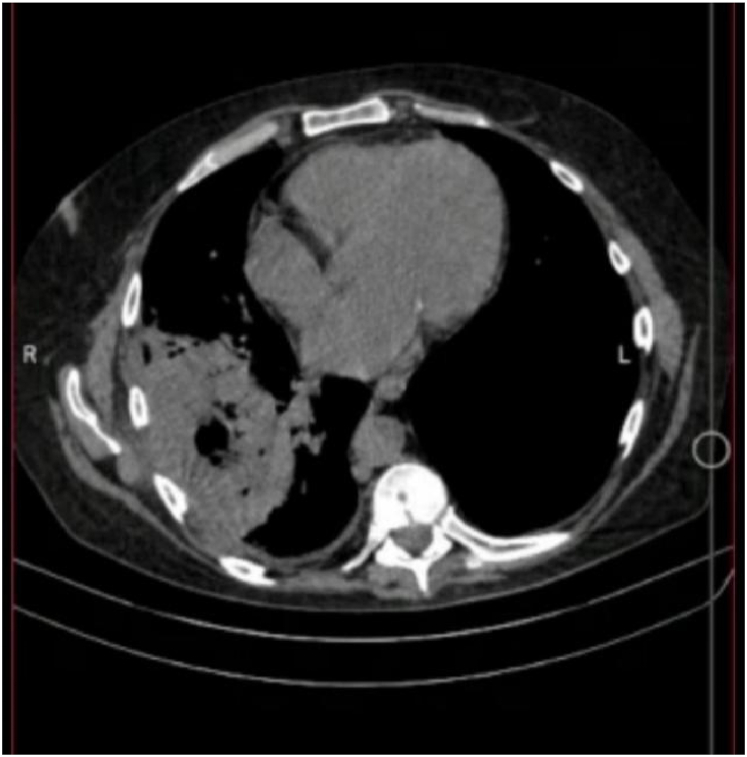
Computerized Topography for the patient chest from the Axial view demonstrating a right lobe cavitary lesion with internal gal bubbles and fluids, measuring about 6.3*5.1*4.8 cm in the right lower lobe associated with adjacent ground glass opacities and minimal left plural and fissural effusion.

**Fig. 2:**
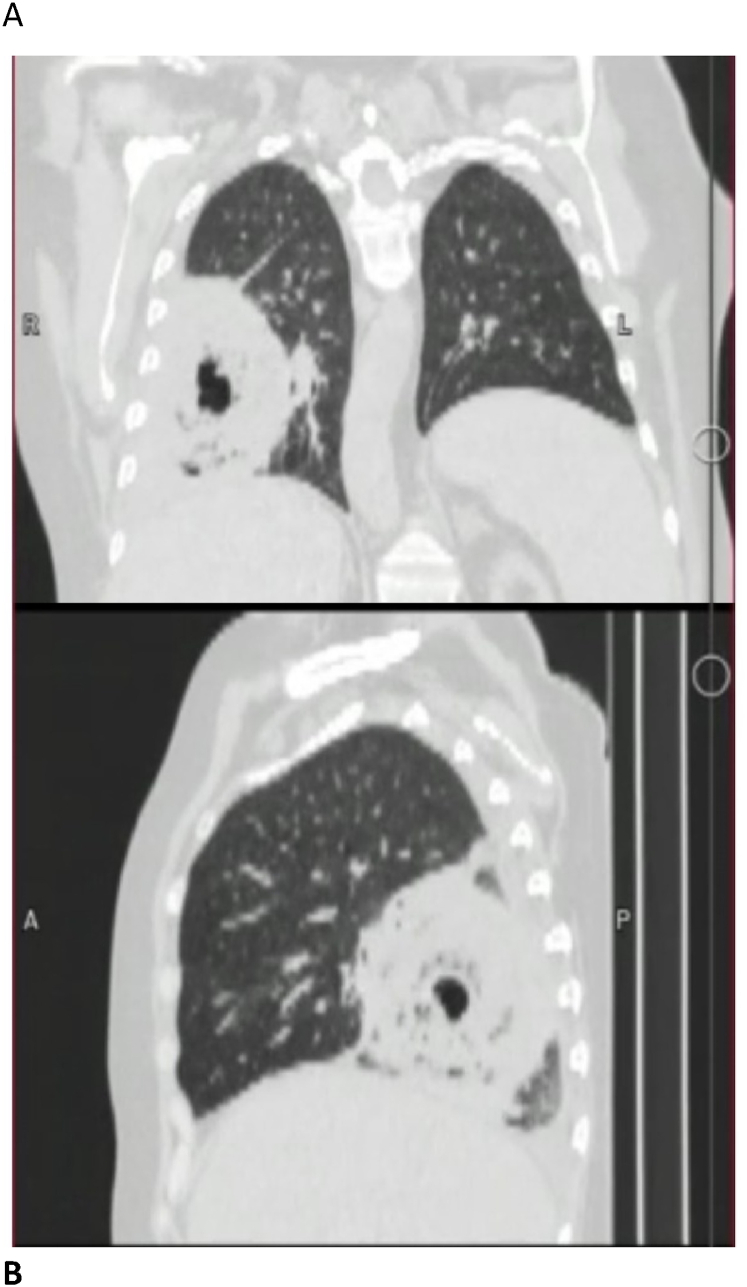
A and B, computerized tomography of the chest from the anterior and lateral views shows right lower lobe cavitary lesion respectively.

His initial chest x ray demonstrated a consolidation in the right lower lobe. Further imaging on chest CT showed a large thick walled cavitary lung lesion with internal gas bubbles and fluids, measuring about 6.3*5.1*4.8 cm in the right lower lobe associated with adjacent ground glass opacities and minimal right plural and fissural effusion (Figs. 1 and 2).

Then, bronchoscopy was performed and bronchoalveolar lavage was obtained from the basal segment of the right lower lobe showed partial bloody thick secretion but no identifiable causative organism. A CT-guided percutaneous drain was inserted into the cavitation and revealed fungal structure that was branching, non septated and variable in width, as evidenced by the Periodic acid–Schiff (PAS) stain, which revealed mucormycosis (see Fig. 4).

As a result, the patient was initiated on amphotericin-B 100mg 1*1 then we proceeded with the surgical option.

**Fig. 3:**
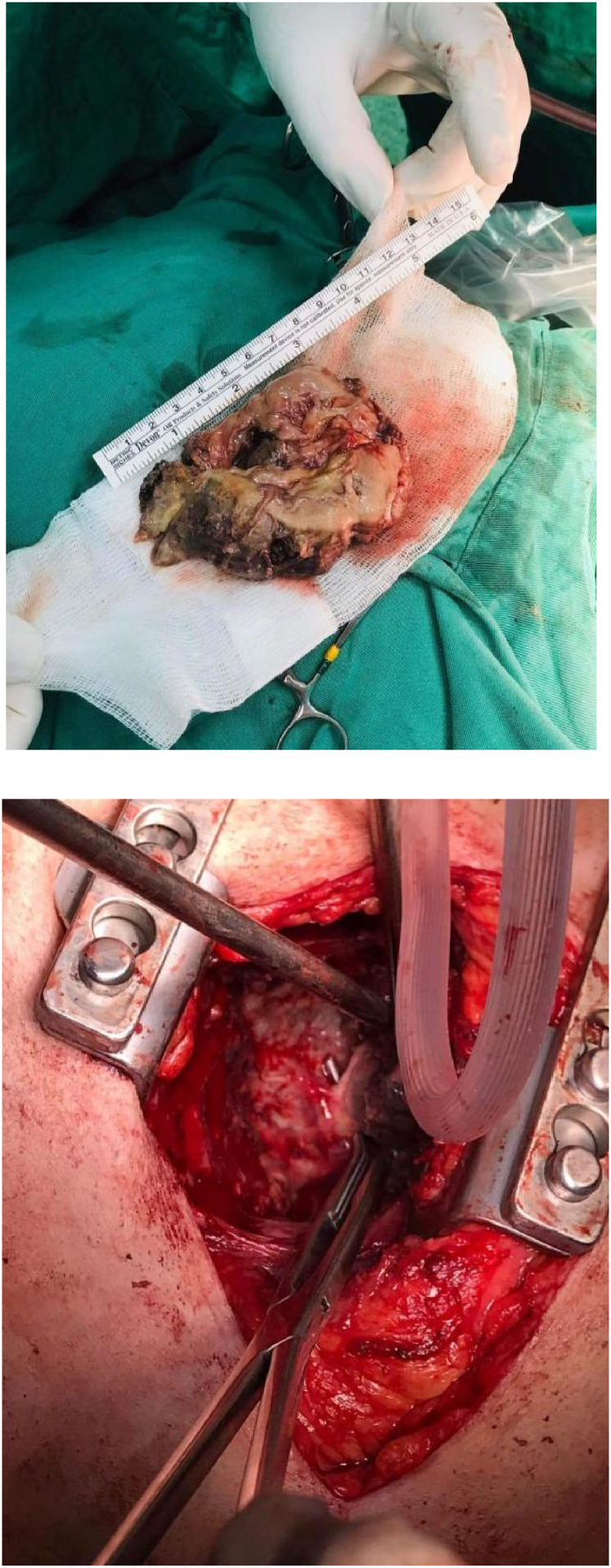
Gross pathology of pulmonary mucormycosis removed from a 46-year-old male patient after renal transplant. (3a) a huge size fungal ball seen at the opening of the lesion. (3b) a large mass removed from the right lower lobe diagnosed as a mass of mucormycosis.

We started by diagnostic bronchoscopy, no endobronchial lesion or fistula were noticed, double lumen intubation was performed, posterolateral thoracotomy in the sixth intercostal space was performed (see Fig. 3), identification of extensive adhesions involving most of the right thoracic cavity, huge fungus ball was identified, removal of the fungus ball using the sharp and blunt dissection, identification of branch of the pulmonary artery and bronchus within the fungus ball, identification of another small bronchial opening involved within the fungus ball, all were dissected and closed under controlled measures. Marsupialization and reinforcing the bronchial opening were performed using 2/0 Vycril absorbable suture, leak test was performed which didn't show any air leak, the cavity left open with one chest tube 28 Fr within it, and another chest tube in the apicoposterior position 28 Fr was inserted. The patient was extubated on table, and continued his medical management.

**Fig. 4:**
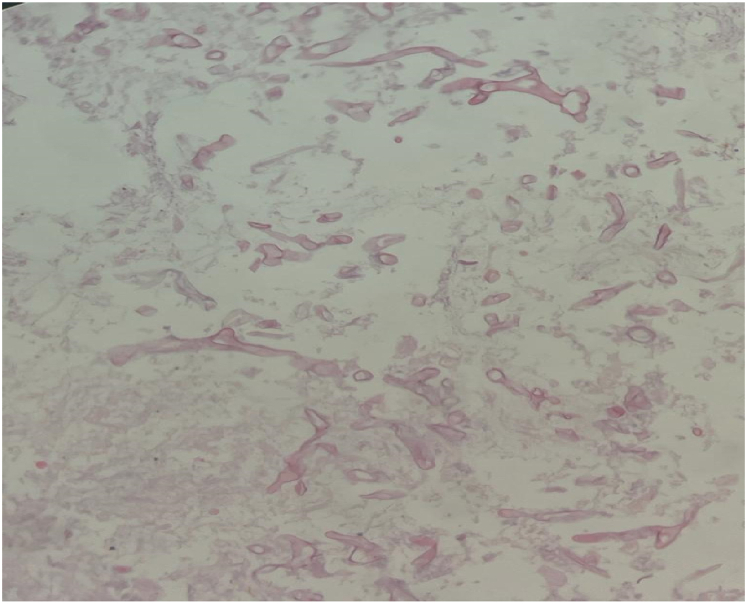
Histopathological examination of the patient's biopsy which was done under CT guided revealed fungal structure that was branching, non septated and variable in width, as evidenced by the Periodic acid–Schiff (PAS) stain, consistent with mucormycosis.

**Fig. 5:**
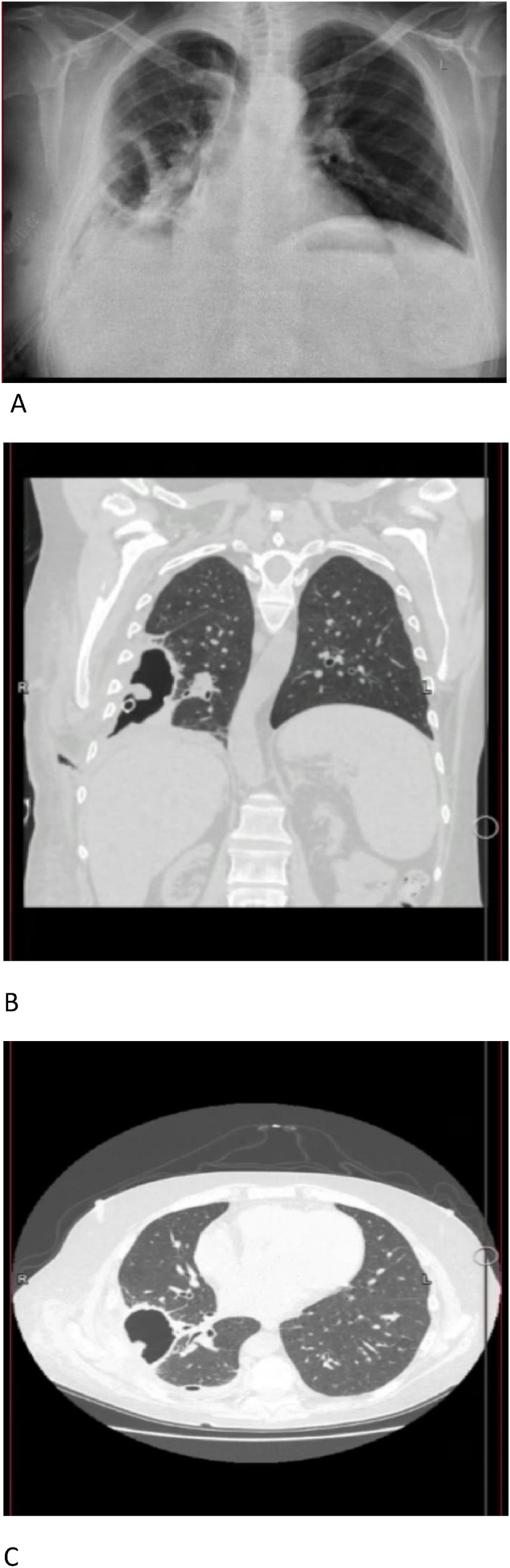
A: Chest x ray. B and C: computerized tomography of the chest from the anterior and transverse views. Postoperative imaging for the patient showing an empty cavity in the right lower lobe and the previous lesion was removed.

Post operatively (see Fig. 5) the patient was doing well with significant improvement of the o2 requirements, improvement of the appetite, with significant improvement of the performance status, the patient was off O2 in the post operative day five, continued on liposomal Amphotericin B three weeks post operatively, follow up CT chest on postoperative day 11 showed no residual disease, no pleural effusion, with residual cavity at the site of removed mucormycosis. The patient was discharged home on post operative day 14 on oral antifungal: Posaconazole, and with chest tube within the cavitary lesion.

## 3 Discussion

### 3.1 Epidemiology and risk factors

Mucormycosis is a rare fungal infection associated with high morbidity and mortality. Rhizopus is the most common species to cause infection followed by mucor in the major genera that causes mucormycosis [6].The main contributing factors are poorly controlled diabetes mellitus both types 1 and 2, diabetic ketoacidosis, glucocorticoid therapy, solid organ transplant [4], hematological malignancies, neutropenia, autoimmune disease, chronic acidemia, iron overload and deferoxamine treatment [6].

In our presented case, the patient is a known case of systemic lupus erythematous, non-diabetic and had renal transplant three months prior to his infection. Invasive fungal infection in SLE patients is very rare and other fungal infections are more common [8].

Mucormycosis after solid organ transplant is related to the use of high doses of corticosteroid after transplant which affect the function of macrophages and neutrophils [9]. In addition, drug induced diabetes is a common side effects for high and persistent use of steroid which enhances the patient's susceptibility for opportunistic infections in general [5].

Although mucormycosis is still a rare complication after renal transplant patients with an incidence of 0.2–1.2%, they are at higher risk for this infection among solid organ transplant patients due to their metabolic disturbances such as uremia and post-transplant diabetes mellitus [10].

Types of mucormycosis are gastrointestinal, pulmonary, cutaneous, rhinocerebral, disseminated and rarely peritonitis in peritoneal dialysis patients. Rhinocerebral mucormycosis is the most common after renal transplant patients followed by pulmonary mucormycosis [5].

Disseminated mucormycosis is defined as infection in two or more noncontiguous sites with a mortality rate near 100%. The risk for dissemination after solid organ transplant depends on the type of that organ with the highest risk after liver transplant 25–55%, the risk for dissemination after renal transplant is 9–13% [2].

### 3.2 Clinical features

Clinical features in patient with mucormycosis depends on the site of infection. For example, rhinocerebral infections present with headache, facial pain, check swelling and eye swelling [11]. pulmonary mucormycosis presents with nonspecific symptoms. Fever is the most prominent presentation, cough, sputum and hemoptysis are common [12]. These nonspecific symptoms may delay the diagnosis of mucormycosis which affect the survival that depends mostly on the early recognition and aggressive management. The cause of death in such patients is related to the spread of the disease, local spread and invasion of the surrounding structures which could lead to massive hemoptysis or due to the angioinvasion and systemic dissemination of the disease [8].

### 3.3 Diagnosis

Early identification of pulmonary mucormycosis might be challenging because it can mimic other prevalent bacterial infections, many patients have a secondary bacterial infection [4] as pulmonary mucormycosis is associated with bacterial pneumonia in 30% of cases, a lack of distinct clinical signs and symptoms and the infection's rarity, virulence, and rapid progression. As a result, they are diagnosed late, which leads to late treatment, and an increased risk of death [13].

Sputum samples are frequently insufficient for diagnosis [8]. Radiographic manifestations are often nonspecific. However high-resolution chest CT can determine the extent of mucormycosis infection earlier than standard chest radiographs [13]. Some studies revealed that the right lung is more commonly involved than the left, and there is a predilection for the upper lobes of the lung because of better aeration, unlike the majority of cases in several studies, our case reported a lesion in the RLL [14].

The gold standard for diagnosis remains a direct histological examination of a tissue specimen [8] via bronchoscopy as it is considered the best diagnostic modality because it offers a relatively safe and minimally invasive method [15]. Our patient's clinical diagnosis of pulmonary mucormycosis was based on the results of a bronchoalveolar lavage (BAL) culture and a biopsy specimen stained with periodic acid-Schiff stain rather than his non-specific symptoms.

There is no serological test available to confirm the diagnosis [13,16]. However Molecular techniques such as polymerase chain reaction (PCR) may allow early and definitive diagnosis in a short period of time, which may improve the outcome [3,8].

Briefly, diagnostic options include clinical and radiographic findings, as well as staining and culture, Identification of characteristic mucoraceus hyphae in injured tissues with right angle branching in a clinical biopsy specimen stained with methanamine silver or periodic acid-Schiff stain is required for definitive diagnosis [13,17,18].

### 3.4 Treatment

Despite the risk of renal toxicity, high-dose liposomal Amphotericin B (5–10 mg/kg/day) [16] is the medication of choice for treating Mucorales spp. Furthermore, all currently available Azole antifungal agents have less effect against mucormycosis; however, itraconazole, isavuconazole and oral posaconazole are used against mucormycosis as alternative in patients with preexisting renal compromise [16] (the incidence of renal injury is significantly lower with isavuconazonium) [4].Voriconazole is not indicated due to the poor susceptibility of Mucorales to this drug [16]. Although some studies have found that combining two antifungals reduces mortality when compared to monotherapy, however these medications are frequently of limited role without surgical intervention (Arana et al., 2021).

In patients with pulmonary mucormycosis, surgical treatment with radical removal of infarcted tissue is the key to improve the results [16] which ought to be done as soon as possible in order to prevent the dissemination [13].

In short, early diagnosis, effective antifungal therapy (IV liposomal amphotericin B is the current gold standard), aggressive surgical debridement with surgical resection of the involved areas of the lung, if necessary, treatment of the underlying disease, and modification of risk factors are all effective management options for pulmonary mucormycosis [8].

### 3.5 Prognosis

Pulmonary mucormycosis has a poor prognosis and outcome that has not improved significantly in the last decade, owing to delayed diagnosis, the type of infection (as pulmonary mucormycosis is a rapidly fatal illness), or the subclass of fungi involved, and the limited activity of current antifungal agents against Mucorales [13,19]. The overall mortality rate of Pulmonary mucormycosis is 76%, increasing to 95% if extrathoracic dissemination occurs. Survival of more than two weeks in the absence of treatment is extremely rare [13].

In summary, we report a rare case of pulmonary mucormycosis in renal transplant recipient with SLE who responded well to intravenous liposomal amphotericin B therapy and surgical debridement. Mucormycosis should be considered as a differential diagnosis in patients with suspected invasive fungal infections and doctors should have high index of suspicion for this rare infection in all immunosuppressed patients to prevent delayed diagnosis and treatment or even misdiagnosis and to be familiar with treatment options in order to improve the prognosis and survival rate in such fatal infection.

### 3.6 Authors recommendations

Mucormycosis is a highly lethal medical condition that occurs more often in immunocompromised patient particularly after solid organ transplantations or prolonged treatment with steroids, our recommendation is to have high level of suspicions when dealing with persistent or growing lesion in these patients despite proper medical therapy, having lower threshold to biopsy these lesions if possible. We recommend to start the proper medical therapy once we suspect this infection, and to implement the surgical option aggressively after confirming the diagnosis, aiming to remove all the infected and necrotic tissue, the prognosis is poor in general but can be improved with early diagnosis and proper management.

## 4 Conclusion

Mucormycosis is a highly lethal complication after kidney transplantation, it could happen at any time during the post-transplant period, has a wide range of clinical manifestations, and is difficult to diagnose. Surgical debridement combined with antifungals (amphotericin B formulation and posaconazole) can enhance a patient's overall survival rate considerably. Clinicians should take special precautions to prevent mucormycosis in renal transplant recipient.

## Ethical approval

The study is exempt from ethical approval in our institution.

## Please state any sources of funding for your research

No funding or grant support.

## Author contribution

Data collection: Dr Yousef Abu Asbeh, Mohammad Eid Mohtasib, Ekhlass abu Asabeh. Study concept or design: Osama N. Dukmak, Yousef abu asbeh. Writing the manuscript: Ekhlass Abu Asabeh, Zahraa zeer, Osama N. Dukmak. Review & editing the manuscript: Osama N. Dukmak, Yousef abu asbeh, Ekhlass Abu asbeh, Zahraa zeer. Histopathological Interpretation: Izzeddin A. Bakri.

## Declaration of competing interest

The authors have no conflict of interests to declare.

## Trial registry number

The study does not have a trial registry number.

## Please state any conflicts of interest

There is no conflict of interest.

## Consent

Written informed consent was obtained from the patient for publication of this case report and accompanying images. A copy of the written consent is available for review by the Editor-in-Chief of this journal on request.

## Registration of research studies

Not applicable.

## Guarantor

Dr. Yousef abu Asbeh.

## Provenance and peer review

Not commissioned, externally peer-reviewed.
